# Two Decades of Coastal Dolphin Population Surveys in Israel, Eastern Mediterranean

**DOI:** 10.3390/biology12020328

**Published:** 2023-02-17

**Authors:** Ori Galili, Oz Goffman, Mia Roditi-Elasar, Yaly Mevorach, Eyal Bigal, Yotam Zuriel, Yaron Haitovich, Nir Hadar, Meytal Markovich, Dror Vardimon, Dana Reininger, Shlomi Marco, Danny Morick, Eliana Ratner, Dan Tchernov, Aviad Scheinin

**Affiliations:** 1 Morris Kahn Marine Research Station, Department of Marine Biology, Leon H. Charney School of Marine Sciences, University of Haifa, Haifa 3498838, Israel; 2 Israel Marine Mammal Research & Assistance Center (IMMRAC), Leon Recanati Institute for Marine Studies, Department of Maritime Civilizations, Leon H. Charney School of Marine Sciences, University of Haifa, Haifa 3498838, Israel; 3 Delphis (NGO), Ashdod 7710202, Israel

**Keywords:** *Tursiops truncatus*, *Delphinus delphis*, Israel, Mediterranean Sea, distribution, conservation, ecology, habitat modeling, Generalized Additive Model

## Abstract

**Simple Summary:**

In the Mediterranean Sea, near the coast of Israel, two dolphin species reside; the common bottlenose dolphin and the common dolphin. These two have been observed by researchers over the last 20 years and have been sighted along different parts of the coast. The two species therefore have different preferences of habitat and in order to evaluate environmental characteristics that are important for each species, statistical models were used to evaluate patterns in their occurrence. Common bottlenose dolphins were found to inhabit the entire length of coast and often chose to swim near fishing trawler vessels. Common dolphins were found to inhabit only the southern section of the Israeli coast and typically chose to swim in shallower waters than the common bottlenose dolphins. It is evident that the two species choose different habitats and the common bottlenose dolphins adapt according to human presence and disturbances, while the common dolphins have more specific preferences that are still not fully understood.

**Abstract:**

Along the Mediterranean coast of Israel, two near-shore dolphin species are prevalent; *Tursiops truncatus* (least concern, IUCN) and *Delphinus delphis* (endangered, IUCN). Ship-board surveys and sporadic sightings over the last two decades have shown that the two differ in distribution—*T. truncatus* is found along the entire coast and *D. delphis* only in the south. The environmental and anthropological factors affecting these species’ spatial distribution and determining their habitat preferences in this area are largely unknown. This work is a first attempt at summarizing 20 years of observations and studying habitat preferences for both species, by use of Generalized Additive Models. *T. truncatus* was found to be present in all areas of the continental shelf where survey effort coverage was sufficient, with a high affinity towards bottom trawlers. Model results showed *D. delphis* distribution to be associated to (shallow) water depths, though the factors driving their limited latitudinal distribution currently remain unknown. It is evident that *T. truncatus* and *D. delphis* are present in segregated areas of the Israeli continental shelf and *T. truncatus* currently sustains a delicate balance with continuously shifting human activities, while the drivers of *D*. *delphis* distribution are more specified, yet still not fully understood.

## 1. Introduction

Across the Levantine Basin in the Mediterranean Sea, only a few studies have been performed on cetacean populations. Although a handful of research cruises have been executed, as well as a singular aerial survey during the ACCOBAMS Survey Initiative, no long-term ecological research has been conducted, with the exception of the Israeli coast. In 1998, IMMRAC (Israel Marine Mammal Research and Assistance Center) began a near-shore research program for coastal dolphins, under the academic umbrella of the University of Haifa. Based on the data that have been collected regularly in Israel on dolphin populations over the last 20 years, it has been established that the Mediterranean coastal shelf along Israel’s shoreline is home to two resident dolphin species—*Tursiops truncatus* (Common bottlenose dolphin) and *Delphinus delphis* (Common dolphin), which are sighted routinely along the shallow continental shelf of Israel and are the focus of this research.

This paper aims to summarize 20 years of dedicated vessel-based dolphin surveys in Israel and outline the main findings on the *T. truncatus* and *D. delphis* populations, their distribution and preferred habitats during this period.

Though both dolphin species are sighted regularly in Israeli Mediterranean waters, *T. truncatus* can be observed along the entire coast and has been documented since surveying began in Israel, while *D. delphis* has been observed anecdotally in 2009 and 2011 near Herzliya and routinely since 2016, in the south of Israel [1]. Worldwide, the *D. delphis* is typically regarded as a pelagic species that feeds on small schooling fish such as anchovies and sardines [2,3], though in the Mediterranean Sea *D. delphis* displays much plasticity in terms of habitat and is found in coastal water and near the shelf edge, in addition to pelagic waters [4,5,6]. This species has also been observed associating with both *T. truncatus* and *Stenella coeruleoalba*, in mixed groups [3,6,7,8]. In Israel, *D. delphis* has been observed exclusively in shallow waters, close to shore and the most common prey item identified from stomach content analysis of stranded individuals is *Ariosoma balearicum*, which is also the most common prey item of *T. truncatus* in Israel and a common by-catch fish in trawler hauls [9].

Abundance of *T. truncatus* has been difficult to gauge and estimations have ranged from 360 [10] to 135 individuals [1]. Alternatively, the local *D. delphis* population is comprised of a distinct cohort, verified by photo-ID analysis, and estimation of abundance is therefore more accurate. Mevorach et al., 2022 [11] has demonstrated that since 2016, when *D. dephis* was initially observed in Israel, this group included up to 37 mature individuals, though since 2020 only the same 15 adults have been observed.

The Levantine basin is the most oligotrophic, saline, and warmest part of the Mediterranean [12]. Seawater temperatures shift between winter lows of 16 °C, to summer highs of 30 °C in a short period of time, effectively resulting in a “Hot Season” (June–November) and a “Cold Season” (December–May) [13]. The Levantine waters near Israel are ultra-oligotrophic [14], the main sources of nutrients being sewage treatment plants, industrial discharge, rivers, the Nile River delta and occasionally Saharan dust formed by aeolian processes [15,16]. Satellite data reveal elevated concentrations of chlorophyll *a* in the vicinity of the Nile River delta extending as far north as the Gaza strip [16,17].

The eastern Mediterranean has been undergoing many changes in recent decades, including shifts in ecosystem composition due to the introduction of invasive species from the Red Sea arriving via the Suez Canal [18,19,20,21,22] and the depletion of local species due to overfishing [23,24,25,26].

Additionally, climate change is a well described phenomenon with various global effects, which is causing measurable increases in water temperature in the eastern Mediterranean (mean of 0.04–0.05 °C per year) [27,28,29] that may potentially alter the spatial and temporal distribution of both the dolphins and their food sources [30].

The Mediterranean trawler fleet of Israel is also an influential and dynamic phenomenon, as the number of active trawlers has decreased throughout the study period from 31 to 15 and the target species have shifted multiple times. Many previous studies have demonstrated *T. truncatus*’ relationship and dependency on trawlers, both in Israel, in other parts of the Mediterranean and worldwide [10,31,32,33,34,35,36]. In Israel, *T. truncatus* have been found to utilize bottom-trawlers and their nets as mobile feeding points [9,37]. Dolphins and *T. truncatus* in particular are opportunistic predators and can shift their dietary preferences, foraging methods and habitat preferences according to available prey [38,39,40].

In Israeli waters, considerable anthropogenic intervention has occurred over the last two decades—establishing Marine Protected Areas, fishing regulations, conducting of seismic surveys, construction of oil and gas facilities, increase in recreational maritime activities and more. All of these activities affect the marine environment and its inhabitants—marine mammals included [41,42,43].

It is unclear how these combined changes will affect *T. truncatus* and *D. delphis* foraging efficiency and strategies as well as distribution. Dolphins, being apex predators, are key indicators of the health of our marine eco-systems [44] and changes in their distribution directly reflect changes in the environment, whether the impact is top-down or bottom-up.

In order to map distribution of cryptic animals, such as marine mammals, habitat models are being applied in many regions worldwide, particularly in areas that are difficult to survey (far offshore) or are heavily impacted by human disturbances [45,46,47]. Modeling populations’ densities, abundance and habitats is advantageous as the models can be used to highlight the main factors that drive a certain species’ distribution and also create prediction maps over un-surveyed areas [45,48]. Determining driving factors and prediction maps can then guide policy makers in creating the appropriate legislative framework for protection of the species [49,50].

## 2. Materials and Methods

### 2.1. Study Area

**Figure 1:**
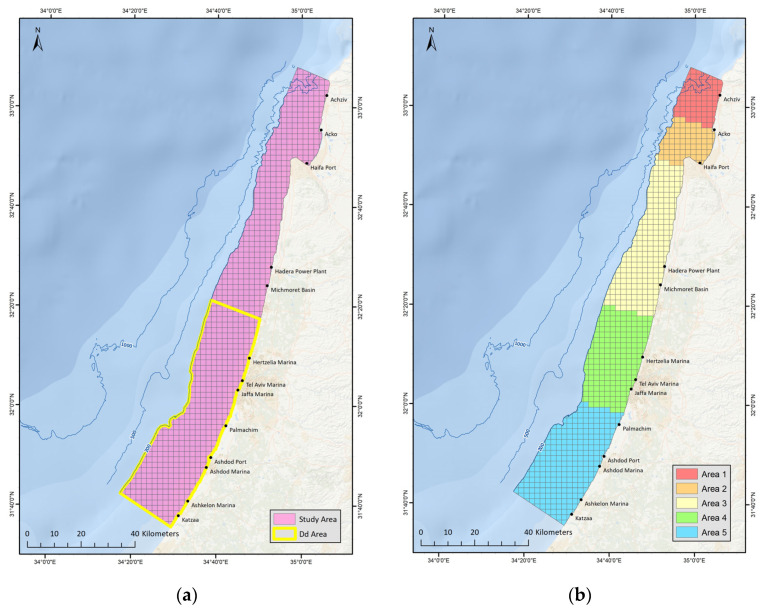
(**a**) A 2 × 2 km gridded map of the study area. Section outlined in yellow indicates the ‘Dd Area’ in the south. Displayed also are the 200 m, 500 m and 1000 m isobars; (**b**) Map displaying division of the continental shelf into 5 spatial subsets.

This study was conducted along the Mediterranean continental shelf of the Israeli coast (Figure 1a). The Mediterranean coastline of Israel, part of the eastern boundary of the Levantine basin, is 196 km long, runs north to south and is virtually featureless, with no significant estuarine rivers. The southern region is characterized by fine sand, while the north by coarser sand and large rock formations [51]. The slope is gradual and in the south the edge of the continental shelf (~100 m depth) can be reached at a distance of approximately 20 km from shore, while in the north the shelf is narrower, approximately 10 km wide [52].

The exact boundaries of the study area are depicted in Figure 1a and this area includes the majority of the survey effort and the majority of sightings for the two dolphin species of interest. The study area’s boundaries were defined by the shoreline, international borders and the 200 m isobar. Although the Achziv sub-sea canyon in the north [53] is deeper than 200 m, this area is near to the shore and has been a significant part of the long-term monitoring and was therefore included in the study area.

### 2.2. Data Collection

Over the last 20 years, researchers from IMMRAC, Delphis (NGO) and the University of Haifa have been conducting half-day nearshore shipboard surveys focused on the coastal dolphin population. For this study, data from the years 1999–2019 were analyzed to study *T. truncatus* distribution and habitat preferences and data from the years 2009–2020 were analyzed to study *D. delphis*.

During the shipboard surveys, the ship’s track is continuously recorded, as well as the sea conditions (wind, wave height, Beaufort Sea State) and information on species, group size, behavior and presence of calves/juveniles. Surveys are initiated only when sea conditions are less than Beaufort Sea State 3, with survey vessels traveling between 7–12 knots and performing a zig-zag search pattern. If a trawler is present near-by, the vessel leaves its planned course in order to search for dolphins that may be feeding around the net. When dolphins are sighted, the vessel follows the group as long as necessary to take photos for individual identification. Although the data input platform has changed between hand-written notes, a designated tablet program and a designated phone app [54], the core information collected and the surveying methodology have remained overall consistent. As surveys were often performed upon opportunistically available platforms, effort was not spread out evenly across the entire study area and optimal search patterns were not always followed.

### 2.3. Habitat Modeling Data

For the habitat modeling portion of this study, the study area was divided into 879 grid-cells, each 2 km × 2 km, in order to create a high-resolution dataset that can differentiate between depths and substrates and can define proximity to various attributes in the marine environment. A sub-section of the study area was utilized during phases of the study relating to *D. delphis* because this species’ extent of occurrence only encompassed Israel’s southern region, which will be referred to from here on as ‘Dd Area’ (Figure 1a).

Datapoints in the dataset were constructed for each occasion that the survey vessel passed within a grid-cell. Each datapoint was then associated with; (a) survey status (active searching/dolphin sighting), (b) dolphin sighting parameters, (number of individuals, behavior), (c) searching type (open-sea search, search near trawler, search near fish-cages) (Table 1), (d) vessel type, (e) Beaufort Sea State (0, 1, 2, 3, 4, 5), (f) environmental parameters.

A large array of environmental data was gathered from multiple sources, as detailed in Table 1. Some of these parameters represent natural variation in the environment (depth, slope, distance from shore, sea surface temperature), some serve as proxies for prey distribution (chlorophyll *a*, bottom content, artificial submerged structures, rigs and fish cages, searching = trawlers, searching = fish-cages), some serve as proxies from anthropogenic effects (distance to rivers, distance to artificial nutrient sources, distance to desalination or power plants) and many also serve as proxies for both prey and anthropogenic effects.

Several considerations were taken into account when obtaining the remote sensing data for ‘sea surface temperature’ and ‘chlorophyll *a’* (as a proxy for primary production), When considering sea surface temperature in the context of this study, the majority of the variability across this parameter occurs over time (between days) and not over space, therefore a data model was used that provided daily means. When considering chlorophyll *a* measurements, the ultra-oligotrophic nature of the study area and high variability require the use of direct measurements. However, many measurements were missing from the data-set due to cloud cover and proximity to shore, causing reduced coverage. This reduced coverage was not able to provide meaningful results during modeling, therefore chlorophyll *a* was ultimately excluded from the modeling process (Supplementary Material, Tables S1 and S2).

### 2.4. Habitat Modeling Approach

Generalized Additive Models were chosen as they are advantageous to modeling natural phenomena as they enable the modeling of complex and nonlinear relationships between the response variable and the predictors. Models were run in ‘R Programming’ with the ‘mgcv’ package [70].

Due to the opportunistic nature of the data collection, the survey effort was not uniform across the study area, and vessel tracks were fragmented according to the 2 km × 2 km grid cells. The result of this high-resolution fragmentation created a dataset that was heavily zero-inflated as well as over-dispersed. In an attempt to cope with the data structure, all models were run twice, once with a Negative Binomial distribution and once with a Tweedie distribution and variables were selected using forward stepwise selection, according to AIC (Supplementary Material, Table S3).

Temporal (ten-year time periods) and seasonal (‘Hot/Cold Season’) subsets were utilized to explore the changes over time and seasonal patterns. Spatial subsets were utilized to explore differences in habitat use across sections of the study delineated by different characteristics with regard to environmental parameters and survey effort (Figure 1b). ‘Area 1’ was adequately surveyed and included the Achziv sub-sea canyon and the vast rocky reefs that are unique to the north of Israel. ‘Area 2’ was inadequately surveyed, only included two dolphin sightings and encompasses the entire Haifa Bay. ‘Area 3’ stretches from Rosh HaCarmel to Hadera and is relatively featureless, with few rocky reefs. ‘Area 3’ was adequately surveyed during the first decade of research, but less so during the second decade. ‘Area 4’ is the most well-surveyed area due to its inclusion of the Herzliya marina, which provided access to a large number of research vessels. ‘Area 4’ is similar to ‘Area 3’, as it is also rather featureless and only includes a few shallow near-shore rocky reefs. ‘Area 5’ is also similar to ‘Area 3’ and ‘4’, as it is mostly featureless, with even fewer rocky reefs and very fine silty sediment. ‘Area 5’ has been adequately surveyed in recent years, though in the past this section has received little survey coverage. Summaries of survey effort in km and number of sightings for the two species can be seen in the Supplementary Material, Tables S4 and S5.

## 3. Results

### 3.1. Survey Results

Between the years 1998 to 2020, 1137 ship-board surveys covered a total of 45,384 km and recorded 346 dolphin sightings. 306 sightings were of *T. truncatus* and 40 sightings were of *D. delphis* (Table 2). *T. truncatus* group sizes ranged between 1–50, with mean group size 5 ± 0.6 (95% CI). *D. delphis* group sizes ranged between 4–30, with mean group size 16.2 ± 5.1 (95% CI). Surveys were carried out year-round, with totaled-surveys-per-year ranging between 6–170 and totaled-sightings-per-year ranging between 2–45.

### 3.2. Distribution

#### 3.2.1. *Tursiops truncatus* Distribution

**Figure 2:**
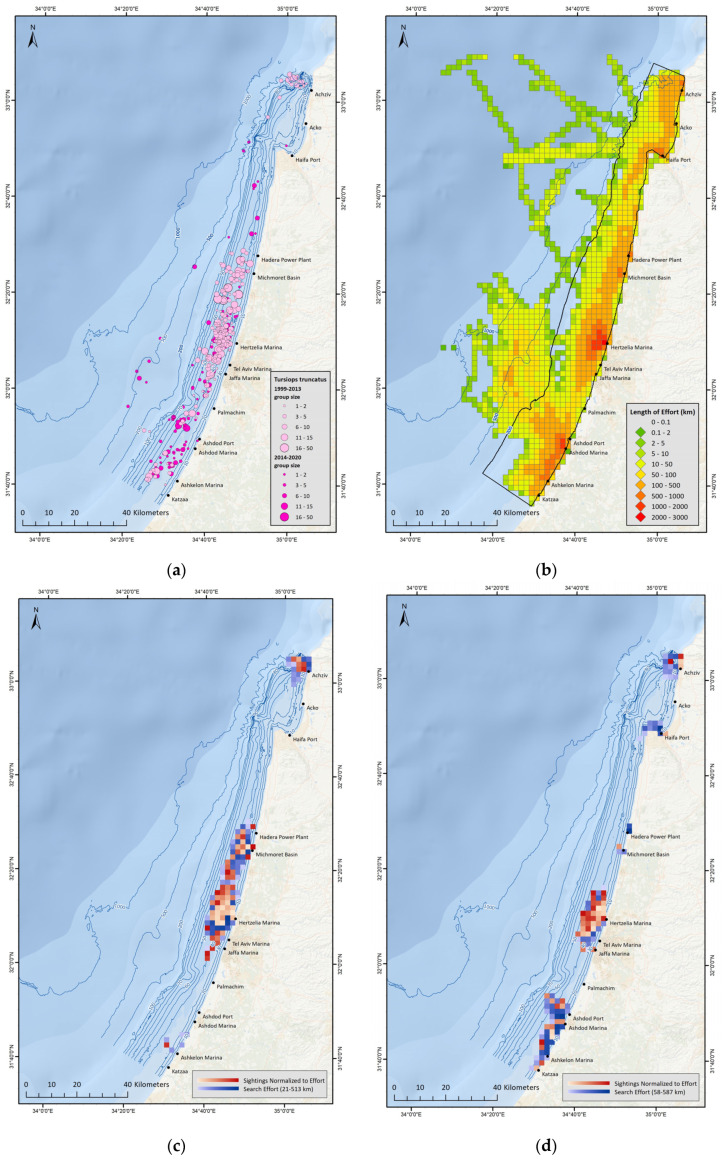
(**a**) Map displaying all sightings from surveys along the Israeli Mediterranean coast between 1999−2020, with the two shades of pink representing *T. truncatus* sightings, before and after the year 2013; (**b**) Map displaying distribution of survey effort between 1999−2020. Each square in the grid represents a 2 km × 2 km area in which total length of vessel tracks across the entire time period was summed; (**c**) Map of study area, displaying survey effort and *T. truncatus* sightings (normalized to survey effort), presenting only grid-cells with sufficient survey effort to surpass the correlation threshold, during the years 1999−2009; (**d**) Map of study area, displaying survey effort and *T. truncatus* sightings (normalized to survey effort), presenting only grid-cells with sufficient survey effort to surpass the correlation threshold, during the years 2010−2019.

Throughout the 20-year study period, all sightings of *T. truncatus* demonstrate that this species is sighted all along the Israeli-Mediterranean coast, in a variety of group sizes, latitudes, depths and distances from shore (Figure 2a). However, the sightings are not entirely uniform across the study area (Figure 2b) and the higher concentration of sightings near Hertzelia are due to higher survey effort in that region, while the lower concentrations around the Haifa Bay are due to lower survey effort in that region. Also, in the north of Israel, around the Achziv sub-sea canyon *T. truncatus* sightings were common up to 2013, after which this species was no longer sighted during designated surveys, with the exception of one sighting in 2020.

In an attempt to correct for the highly un-uniform spatial distribution of survey effort, subsets were created that only included grid-cells in which the occurrence of dolphin observations was not statistically correlated to search effort. Utilizing ‘Spearman’s Rank Correlation Test’, a threshold relating to ‘minimum survey effort per grid-cell’ was determined. Only grid-cells containing accumulative survey effort (total km) that exceeded the threshold were retained in these subsets (Figure 2c,d). Overall, percentage of data retained from the entire data set varied between 33–42% and percentage of sightings retained varied between 31–37%.

#### 3.2.2. *Delphinus delphis* Distribution

**Figure 3:**
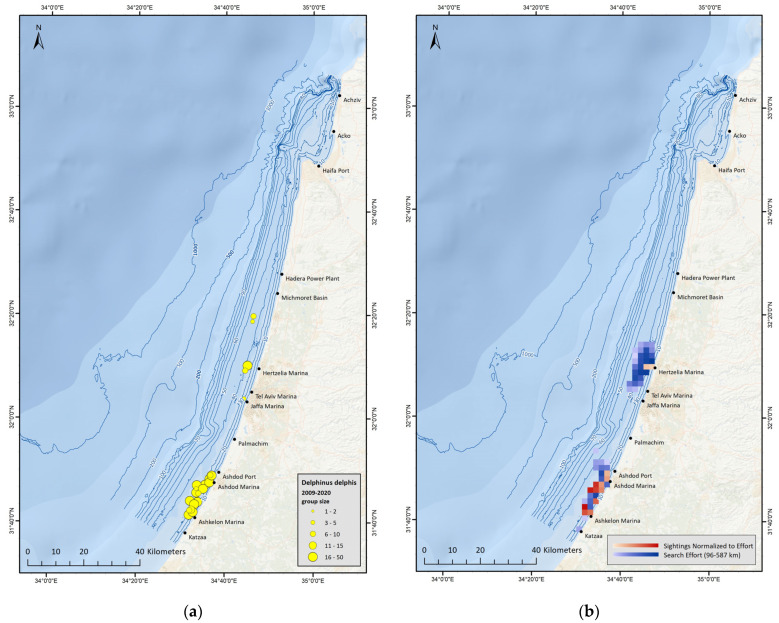
(**a**) Map displaying all sightings from surveys along the Israeli Mediterranean coast between 1999−2020, with the yellow circles representing *D. delphis* sightings; (**b**) Map of study area, displaying survey effort and *D. delphis* sightings (normalized to survey effort), presenting only grid-cells with sufficient survey effort to surpass the correlation threshold, during the years 2010−2019.

*D. delphis* are sighted regularly in Israel during recent years, mostly south of Ashdod, in large groups (15–30 individuals), including calves [11,71]. To date, all reported *D. delphis* sightings in the area were in shallow water, at depths less than 40 m (Figure 3a), a finding also strengthened by results of ‘Spearman’s Rank Correlation Test’ (Figure 3b). In addition, *D. delphis* presence appears to show seasonality, with no sightings from designated surveys during the months of January, February and March, although survey effort was sufficient during this time period.

### 3.3. Habitat Models

#### 3.3.1. *Tursiops truncatus* Habitat Models

The most predominant explanatory variable across all *T. truncatus* models (Supplementary Material, Table S3) was the ‘Searching’ variable, which indicated that *T. truncatus’* probability of occurrence was higher when the survey vessel searched for dolphins in the vicinity of trawlers rather than open water. Additionally, ‘Distance to Desalination or Power Plants’ was significant throughout many of the models, though the likeliest explanation for this result is attributed to the proximity of these facilities to the marinas, which provided the start and end points for the majority of surveys. Lastly, ‘Sea Surface Temperature’ was significant across multiple models, though trends displayed in the plots were highly variable and, in many cases, may have presented overfitting of the data.

Across the temporal subsets, four parameters were significant for both Negative Binomial and Tweedie models— ‘Searching’, ‘Distance to Desal or Power Plants’, ‘Distance to Shore’ and ‘Sea Surface Temperature’ (Supplementary Material, Table S6).

Modeling results from the seasonal models showed that the only parameter that was significant in all models based on the ‘Hot Season’ dataset was the ‘Searching’ parameter, again demonstrating that dolphins associate strongly with trawlers. Comparison between the Negative Binomial models and the Tweedie models show similar results, though the Tweedie models found significance in fewer parameters. Modeling results based on the ‘Cold Season’ dataset, did not find parameters that were significant for all models. Overall, dolphin distribution and habitat preferences appear unpredictable during the ‘Cold Season’. Comparison between the Negative Binomial models and the Tweedie models shows differing results across ‘Cold Season’ models, both in terms of significant parameters and trends observed for the parameters retained (Supplementary Material, Tables S7 and S8).

Models from ‘Area 1’ resulted in a lot of uncertainty across most variables though some preference is observed towards trawlers in that area, which today are no longer permitted by law. Models from ‘Area 2’ did not include enough sightings for valid results. Models from ‘Area 3’ resulted in a lot of uncertainty across most variables and some preference towards trawlers. Models from ‘Area 4’ resulted in a lot of uncertainty across most variables and a definite preference towards trawlers. Models from ‘Area 5’ resulted in a lot of uncertainty across most variables and some preference towards trawlers and the offshore fish cages (Supplementary Material, Table S9).

All model results indicated that the location and presence of trawlers are the main driving factors for *T. truncatus* distribution. In an attempt to assess distribution drivers in the absence of trawlers, an additional subset was created, which excluded all data (search effort and sightings) that was associated with presence of a trawler. Prediction maps were created based on four different models from the ‘trawler excluded’ subset and these display the probability of occurrence for *T. truncatus* along the Israeli continental shelf, according to the findings of each model (Supplementary Material, Tables S10 and S11).

**Figure 4:**
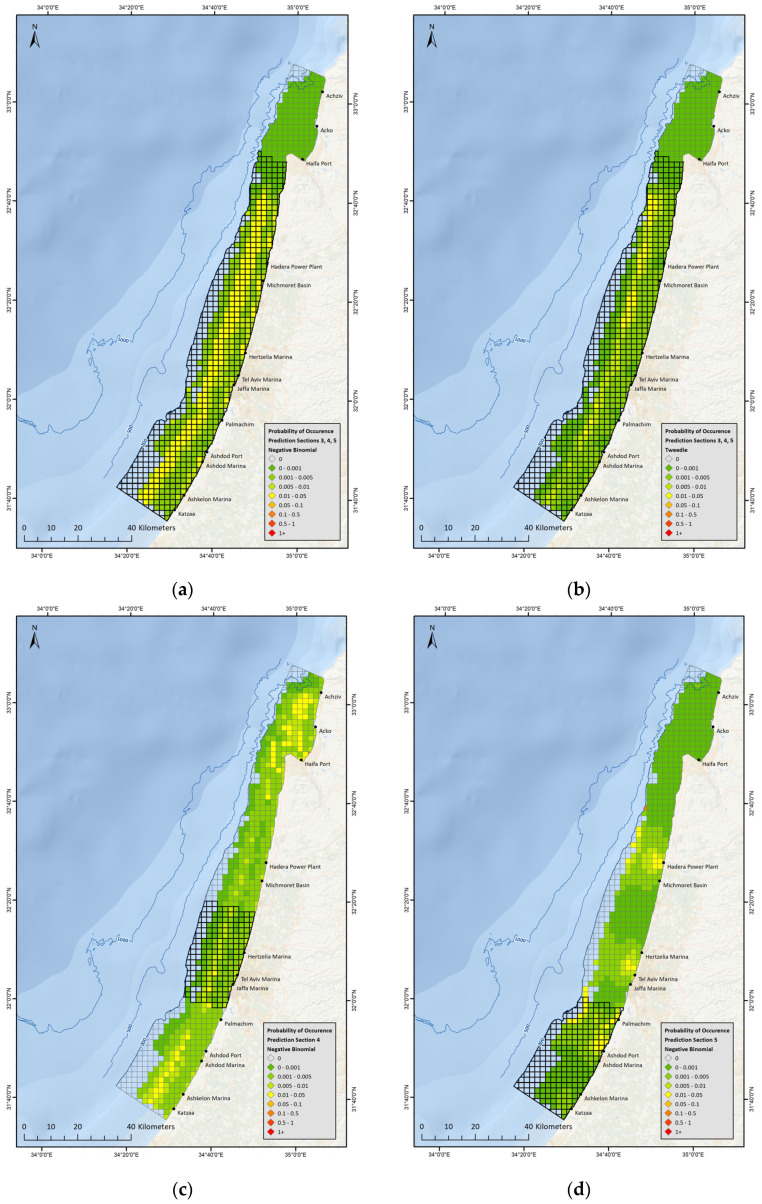
(**a**) Prediction Map for *T. truncatus* based on Negative Binomial distribution and ‘trawler excluded’ data from Areas 3, 4 and 5; (**b**) Prediction Map for *T. truncatus* based on Tweedie distribution and ‘trawler excluded’ data from Areas 3, 4 and 5; (**c**) Prediction Map for *T. truncatus* based on Negative Binomial distribution and ‘trawler excluded’ data from ‘Area 4’; (**d**) Prediction Map for *T. truncatus* based on Negative Binomial distribution and ‘trawler excluded’ data from ‘Area 5’.

The prediction maps produced a visual display that reflects the environmental variables affecting the final prediction. Across these four models, certain explanatory variables had a stronger effect on the final prediction, such as ‘Depth’ (Figure 4a–c) or ‘Distance to desalination or power plants’ (Figure 4d). The maps also display an overall similarity when comparing between predictions utilizing the Negative Binomial distribution or the Tweedie distribution on subsets from ‘Areas 3’, ‘4’ & ‘5’ combined, (Figure 4a,b).

#### 3.3.2. *Delphinus delphis* Habitat Models

As *D. delphis* have only been sighted in the south of Israel and in recent years, three spatio-temporal subsets were created for modeling their distribution (Supplementary Material, Tables S12 and S13). Models run according to the Negative Binomial distribution presented a varied number of explanatory variables, between 2–5. ‘Depth’, ‘Distance to Shore’ and ‘Searching’ were the three variables that were retained in more than one model. All three models run according to the Tweedie distribution, only retained ‘Depth’ as the singular explanatory variable.

## 4. Discussion

### 4.1. Tursiops truncatus

Survey observations as well as visualized prediction maps derived from ‘non-correlated data’ models demonstrate that *T. truncatus* is sighted along the entire continental shelf in all areas where surveys were conducted. *T. truncatus* has a notable affinity towards trawlers—an observation which is also supported by habitat model results. Since 2016 fishing regulations have limited trawling operations, and all areas north of Dor (mid ‘Area-3’) are prohibited for trawling year-round, in addition to a seasonal no-trawling period along the entire coast in July and August. Due to the relative novelty of these regulations, there is not enough data to determine how the change in trawling patterns has affected *T. truncatus* distribution.

In the north of Israel, near Rosh Hanikra and the Achziv sub-sea canyon, *T. truncatus* sightings from designated surveys became scarce from the year 2014 and onwards. This finding may be related to an existing acoustic disturbance in the north in recent years, though this speculation has yet to be proven with certainty. Additionally, sightings reported from the general public are also very rare between Akko and Achziv in recent years. Either way, it appears that *T. truncatus* currently displays a preference towards the area of the Israeli coast from the Haifa Bay and southwards.

The difference found across models from the ‘Hot Season’ and ‘Cold Season’ in regard to association with trawlers may be attributed to: (1) Differences in trawler patterns, (2) Differences in prey availability in the natural environment and therefore differences in foraging strategies, (3) Differences in energetic budgets between seasons, resulting in differences attributed to foraging strategies. Trawl haul has been shown to change across the seasons [72], with recorded differences in total abundance, biomass and species composition. In all categories, significant differences were found between summer hauls, presenting higher values in comparison to other seasons, which varied across the different categories examined. This observation demonstrates that it is therefore possible that foraging trawler nets is more rewarding to *T. truncatus* during the ‘Hot Season’.

Prediction maps based on modeling results indicated that *T. truncatus* display a preference towards a corridor, approximately 3–5 km wide, that runs parallel to shore, at a distance of 6–8 km. This spatial preference could be related to depth, distance from shore (both as determinants of prey concentration), or following typical trawling routes in anticipation of encounters, and currently there is not enough data to discriminate between these possibilities.

### 4.2. Delphinus delphis

Survey observations conclude that *D. delphis* are sighted only in the south of Israel, between ‘Palmachim’ and ‘Ashkelon’ and only in shallow waters between 20–40 m depth. Model results also support these observations, as ‘Depth’ was found to be the most significant environmental parameter for *D. delphis* distribution. These findings do not fully align with previous findings from stomach content analysis, which indicate an association between *D. delphis* and trawlers. Furthermore, throughout designated surveys, *D. delphis* were only occasionally observed near trawlers, mainly while in their sorting process, and it is possible that the few individuals that were stranded (deceased) are not a good representation of the entire population but rather represent outliers that died of unnatural causes.

Considering the limited survey effort in deep waters, as well as the variability in this species’ observed habitat in the Mediterranean Sea, and the fact that *D. delphis* tends to inhabit deeper water than *T. truncatus* in overlapped areas around the world and in the Mediterranean Sea in particular [2,5,73], it is not possible to rule out the option that there may be some pelagic presence of *D. delphis* in Israel as well.

Although according to survey results this species display a seasonal trend of absence during winter, 10% of *D. delphis* sightings reported by the general public since 2015, occurred during the months of January-March, demonstrating that *D. delphis* is not completely absent during these months, though less prevalent. It should also be noted that these three months are some of Israel’s coldest and stormiest winter months, making surveying difficult and minimizing the time the general public also spends out at sea.

Lastly, it should be noted that nearly all surveys in this region were conducted during the day, with the vast majority of surveys setting out in the morning and ending by noon, therefore it is also possible that *D. delphis’s* spatial distribution is affected by diurnal patterns overlooked by the data. Small delphinids, including *D. delphis* have been documented to exhibit changes in behavior and distribution (nearshore vs offshore waters) across different hours of the day [74,75,76].

This species is considered ‘Threatened’ in the Mediterranean [77] and the small population observed along Israel’s southern coast appears to be highly localized. Results of Photo ID analysis revealed that the individuals in the south of Israel are part of one distinct cohort and do not mix with other groups in the region. This cohort is restricted to less than 20 mature individuals [11], which by numbers alone warrants a ‘critically endangered’ status.

## 5. Conclusions

Two decades of designated surveys have shown that sightings of *T. truncatus* occur across the entire length of Israel’s coastline while *D. delphis* sightings are clustered and confined to the south region. Although the restricted distribution of *D. delphis* may be related to higher concentrations of nutrients from the Nile River delta, this speculation could not be confirmed due to constraints in the collection of environmental data. Although survey observations and model results were not robust enough to detect temporal or spatial trends related to industrial anthropogenic effects, habitat models did provide partial answers to differences in distribution between the two species, with their results matching survey observations, demonstrating that *D. delphis* prefers shallower depths and *T. truncatus* prefers association with trawlers and fish farms.

Across all maps created during this study, it is evident that *T. truncatus* and *D. delphis* are present in segregated areas of the Israeli continental shelf. Due to the distinct spatial separation (according to depth) and different foraging strategies presented by the two species in the south of Israel, it is likely that their co-existence in the region is made possible by niche separation, though additional research on the topic is currently underway. Furthermore, there are no recorded observations of mixed species groups or interactions between the two species.

## Figures and Tables

**Table 1:** Environmental parameter names, sources and methods of analysis.

Environmental Parameter	Data Source ^1^
Searching (type)	Searching status during survey effort: open sea searching/near trawler/near fish cages
Depth	GEBCO Compilation Group 2020 [55]
Slope	GEBCO Compilation Group 2020 [55]
Distance from Shore	Country border shapefiles from DIVA-GIS website [56]
Distance to Rivers	River delta locations as displayed in World Oceans Basemap [57]. Rivers referenced in this dataset are based on IOLR Monitoring Reports [58,59,60,61] found in the Ministry of Environmental Protection website [62]
Distance to Artificial Nutrient Sources	Data based on pipelines permitted to release organic waste into the marine environment according to the Ministry of Environmental Protection [63]
Distance to Power Plants & Desalination Plants	Data based on pipelines permitted to release brine or coolant water into the marine environment according to the Ministry of Environmental Protection [63]
Artificial Submerged Structures	Locations of shipwrecks and other unidentified wreckage was obtained from local fishermen and divers
Rigs and Fish cages	Locations of three gas rigs and two offshore fish cages are included in this dataset
Bottom Content	Type of bottom content: sand/mud/rock Data was obtained thanks to the assistance of the Geological Survey of Israel [64,65,66,67]
Sea Surface Temperature	Remote sensing date from the E.U. Copernicus Marine Service Information website [68]
Chlorophyll *a*	Remote sensing date from the E.U. Copernicus Marine Service Information website [68]

^1^ All data was mapped, processed and measured in ArcGIS [69].

**Table 2:** Table displaying the number of kilometers surveyed across the entire survey area and across the Dd Area per year, as well as number of *T. truncatus* and *D. delphis* sightings per year.

Year	Total km across Survey Area	*T. truncatus* Sightings	Total km across Dd Area	*D. delphis* Sightings
1999	335	4	77	0
2000	526	6	63	0
2001	202	3	75	0
2002	273	8	206	0
2003	629	5	444	0
2004	1320	11	621	0
2005	1435	21	1143	0
2006	1881	18	1729	0
2007	1202	11	1010	0
2008	1173	14	738	0
2009	1938	18	1117	2
2010	1934	34	1310	0
2011	2548	24	1852	2
2012	1822	21	1416	0
2013	1470	18	855	0
2014	1212	12	964	0
2015	710	6	597	0
2016	1245	6	969	3
2017	4894	24	2167	1
2018	4099	16	2084	6
2019	8803	28	5222	8
2020	5733	30	3565	18

## Data Availability

Not applicable.

## Supplementary Materials

The following supporting information can be downloaded at: https://www.mdpi.com/article/10.3390/biology12020328/s1, Table S1: Model comparison of three main models; (1) All Data, (2) 1999–2009 and (3) 2010–2019, for datasets with and without ‘chlorophyll *a*’ data; Table S2: Models for *Tursiops truncatus* distribution, including the variable ‘chlorophyll *a*’ as run on the full dataset for all years, years 1999–2009 and years 2010–2019; Table S3: Summary of final models for each sub-set. Information includes number of sightings within the subset, deviance explained for Negative Binomial based models and Tweedie based models and names of explanatory variables for Negative Binomial based models and Tweedie based models; Table S4: Table displaying total kilometers of survey effort between the years 1999–2020 across the entire study area as well as across the ‘Dd Area’. Also displayed is the number of *T. truncatus* and *D. delphis* sighting. Each parameter is summed according to month-of-the-year and displayed according to relevant month; Table S5: Table displaying total kilometers of survey effort between the years 1999–2020 across the five sections of the study area as well as across outside the study area. Also displayed is the number of *T. truncatus* and *D. delphis* sighting in each area; Table S6: Final models for *Tursiops truncatus* distribution, as run on the full dataset for all years, years 1999–2009 and years 2010–2019; Table S7: Final models for *Tursiops truncatus* distribution, as run on the ‘Hot Season’ dataset for all years, years 1999–2009 and years 2010–2019; Table S8: Final models for *Tursiops truncatus* distribution, as run on the ‘Cold Season’ dataset for all years, years 1999–2009 and years 2010–2019; Table S9: Final models for *Tursiops truncatus* distribution, as run on the spatial subsets (Are½1/2/3/4/5) for all years, years 1999–2009 and years 2010–2019; Table S10: Summary of ‘trawler-excluded’ models for each spatial section. Information includes number of sightings within the subset, number of zero values, deviance explained for both Negative Binomial and Tweedie models and names of explanatory variables for both Negative Binomial and Tweedie models; Table S11: Final models for *Tursiops truncatus* distribution, as run on the ‘trawler-excluded’ dataset (Areas 3 & 4 & 5/3/4/5) for all years, years 1999–2009 and years 2010–2019; Table S12: Summary of models from the three *Delphinus delphis* subsets. Information includes number of sightings within the subset, number of zero values, deviance explained for both Negative Binomial and Tweedie models and names of explanatory variables for both Negative Binomial and Tweedie models; Table S13: Final models for *Delphinus delphis* distribution, as run on three subsets.
