# Supplementary material for: Two Decades of Coastal Dolphin Population Surveys in Israel, Eastern Mediterranean
Source: Biology (Basel). 2023 Feb 17;12(2):328. doi: 10.3390/biology12020328 (PMC9952934; doi:10.3390/biology12020328)
Supplement: Supplementary file 1 [file biology-12-00328-s001.zip › biology-2158956-supplementary.pdf]

Table S1. Model comparison of three main models; 1) All Data, 2) 1999-2009, and 3) 2010-2019, for datasets with and without 'chlorophyll *a*' data.

Model name	N dolphin observations	N Zero	% non zero	N variables	% Deviance Explained	AIC	Variable names
All Years CHL	252	2062	12.22	6	21	2786	DESAL + SEARCHING + SHORE + SST + BOTTOM + SLOPE
1999-2009 CHL	99	1681	5.89	8	25	1223	SEARCHING + SHORE + DEPTH + DESAL + WRECK + SLOPE + SST + CHL
2010-2019 CHL	153	1737	8.81	5	26	1775	DESAL + SST + SHORE + SEARCHING + DEPTH
All Years	284	2362	12.02	7	23	3134	DESAL + SEARCHING + SHORE + DEPTH + SST + BOTTOM + SLOPE
1999-2009	113	1850	6.11	8	23	1391	SEARCHING + SHORE + DESAL + DEPTH + WRECK + SLOPE + BOTTOM + SST
2010-2019	171	2028	8.43	6	31	1988	DESAL + SST + SHORE + SEARCHING + DEPTH + NUTRIENTS

.

Table S2. Models for *Tursiops truncatus* distribution, including the variable ‘chlorophyll *a*’ as run on the full dataset for all years, years 1999-2009, and years 2010-2019. First three rows display results utilizing Negative Binomial distribution, while following three rows utilize Tweedie distribution. Variables that are statistically significant have names marked in blue. All vertical axes indicate the probability of dolphin occurrence on a scale of 0 to 1 (except instances where scale is minimized for clarity), while horizontal axes are presented in the units of the relevant explanatory variable, and above the horizontal axis are markings, indicating number of non-zero observations at that value of explanatory variable. Gray shaded areas represent 95% Confidence Interval.

Negative Binomial Distribution, Chlorophyll *a* not incuded								
All Years Desal	All Years Shore	All Years Depth	All Years SST	All Years Slope		**All Years Searching**	All Years Bottom	
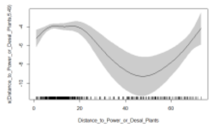	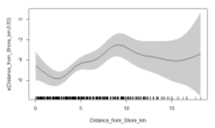	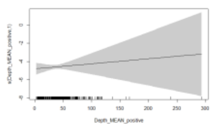	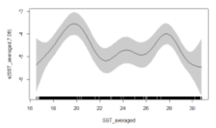	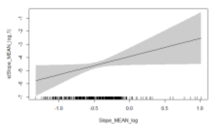		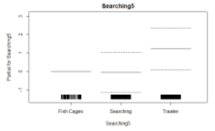	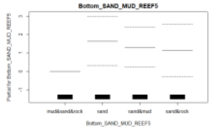	
1999-2009 Desal	1999-2009 Shore	1999-2009 Depth	1999-2009 SST	1999-2009 Slope		**1999-2009 Searching**	1999-2009 Bottom	1999-2009 Structure
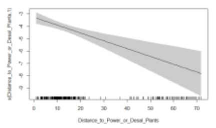	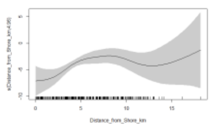	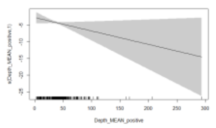	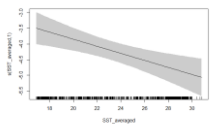	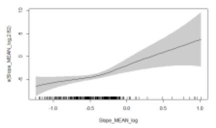		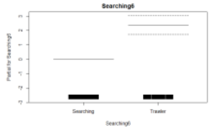	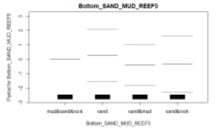	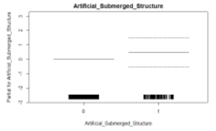
2010-2019 Desal	2010-2019 Shore	2010-2019 Depth	2010-2019 SST		2010-2019 Nutrients	**2010-2019 Searching**		
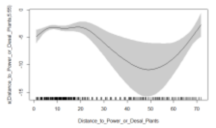	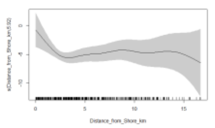	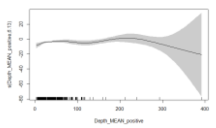	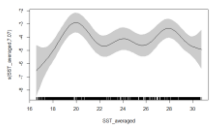		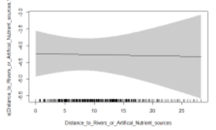	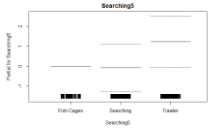		
Negative Binomial Distribution, Chlorophyll *a* included in dataset								
All Years Desal	All Years Shore		All Years SST	All Years Slope		**All Years Searching**	All Years Bottom	
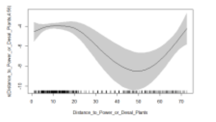	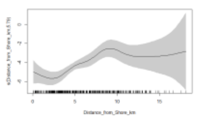		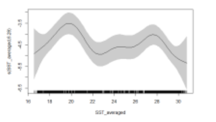	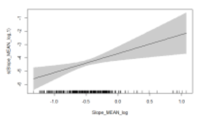		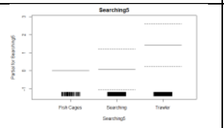	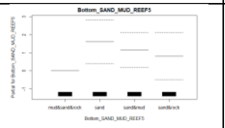	
1999-2009 Desal	1999-2009 Shore	1999-2009 Depth	1999-2009 SST	1999-2009 Slope	1999-2009 Chlorophyll	1999-2009 Searching		1999-2009 Structure
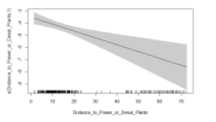	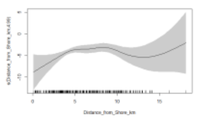	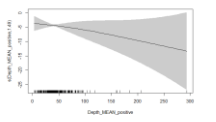	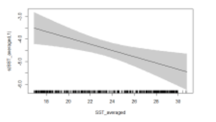	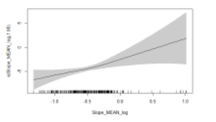	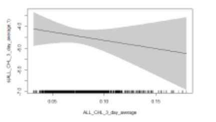	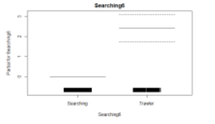		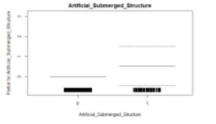
2010-2019 Desal	2010-2019 Shore	2010-2019 Depth	2010-2019 SST			**2010-2019 Searching**		
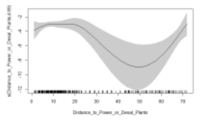	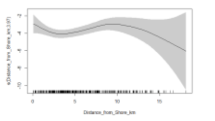	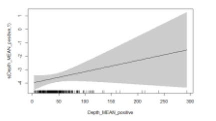	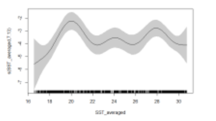			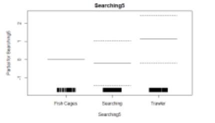		

Table S3. Summary of final models for each sub-set. Information includes number of sightings within the subset, deviance explained for Negative Binomial based models and Tweedie based models, and names of explanatory variables for Negative Binomial based models and Tweedie based models.

Model name	N Dolphin Observations	N Zeros	% Non-Zero	% Deviance Explained (NB)	Variable names (NB)	% Deviance Explained (TW)	Variable names (TW)
All Years	284	2362	12	23	DESAL + SEARCHING + SHORE + DEPTH + SST + BOTTOM + SLOPE	21	DESAL + SEARCHING + SLOPE + DEPTH + SST + NUTRIENTS + WRECK
1999-2009	113	1850	6.1	23	SEARCHING + SHORE + DESAL + DEPTH + WRECK + SLOPE + BOTTOM + SST	18	SEARCHING + SST + SHORE + DESAL + SLOPE + DEPTH
2010-2019	171	2028	8.4	31	DESAL + SST + SHORE + SEARCHING + DEPTH + NUTRIENTS	26	DESAL + SHORE + SEARCHING + SST
All Years HOT	154	2521	6.1	26	SEARCHING + DESAL + SST + SHORE + BOTTOM	27	SEARCHING + DESAL + BOTTOM
All Years COLD	130	2204	5.9	27	DESAL + SHORE + SST + SEARCHING + SLOPE + NUTRIENTS + WRECK	23	DESAL + DEPTH + SEARCHING + SST
1999-2009 HOT	61	2010	3	39	SEARCHING + SHORE + SST + WRECK + NUTRIENTS	16	SEARCHING
1999-2009 COLD	52	1691	3.1	18	DESAL + SHORE + WRECK + SEARCHING + SLOPE + NUTRIENTS + BOTTOM	05	SEARCHING + WRECK
2010-2019 HOT	93	2146	4.3	29	DESAL +SEARCHING + SHORE + SST + BOTTOM + DEPTH	22	DESAL +SEARCHING
2010-2019 COLD	78	1909	4.1	36	DESAL + SST + SEARCHING + NUTRIENTS + WRECK	34	DESAL + SHORE + DEPTH + SEARCHING
All Years Area 1	13	739	1.8	46	DESAL	44	DESAL + SEARCHING
All Years Area 2	1	NA	NA	NA	NA	NA	NA
All Years Area 3	43	1449	3	40	SEARCHING + DESAL + BOTTOM + DEPTH	33	DESAL + SEARCHING
All Years Area 4	172	3207	5.4	15	SST + SEARCHING + SHORE	10	SEARCHING + SST
All Years Area 5	55	2009	2.7	07	SEARCHING	5	SEARCHING
1999-2009 Area 1	5	232	2.2	38	SST	28	SLOPE + DEPTH
1999-2009 Area 2	1	NA	NA	NA	NA	NA	NA
1999-2009 Area 3	35	663	5.3	23	SEARCHING + BOTTOM + WRECK	21	SEARCHING + DEPTH
1999-2009 Area 4	65	1362	4.8	18	SEARCHING + SST	11	SEARCHING
1999-2009 Area 5	7	168	4.2	31	SST	33	SST
2010-2019 Area 1	8	507	1.6	74	DEPTH + DESAL + SEARCHING	50	DESAL + SEARCHING
2010-2019 Area 2	0	NA	NA	NA	NA	NA	NA
2010-2019 Area 3	8	786	1	72	SST + BOTTOM	18	SLOPE
2010-2019 Area 4	107	1845	5.8	15	SST + SHORE + DEPTH + SEARCHING	7	DESAL + SEARCHING
2010-2019 Area 5	48	1842	2.6	10	SEARCHING	1	SST

Table S4. Table displaying total kilometers of survey effort between the years 1999-2020 across the entire study area as well as across the 'Dd Area'. Also displayed is the number of *T. truncatus* and *D. delphis* sighting. Each parameter is summed according to month-of-the-year and displayed according to relevant month.

1999-2020	Jan	Feb	Mar	Apr	May	Jun	Jul	Aug	Sep	Oct	Nov	Dec
Study Area - Kilometers Surveyed	3,022	3,123	2,453	2,722	5,993	6,949	4,100	4,525	3,133	2,922	3,674	2,768
T. truncatus Sightings	27	19	15	31	30	37	24	34	29	35	33	24
Dd Area - Kilometers Surveyed	1,703	1,960	1,439	1,930	3,384	3,511	2,617	3,199	2,070	2,087	2,452	1,869
D. delphis Sightings	0	0	0	1	6	6	10	6	3	3	3	2

Table S5. Table displaying total kilometers of survey effort between the years 1999-2020 across the five sections of the study area as well as across outside the study area. Also displayed is the number of *T. truncatus* and *D. delphis* sighting in each area.

1999 - 2020	Area 1	Area 2	Area 3	Area 4	Area 5	Outside study area
Total Kilometers Surveyed	3,982	2,837	6,830	15,099	12,386	4,114
T. tursiops Sightings	18	3	47	178	80	12
D. delphis Sightings	0	0	1	4	35	0

Table S6. Final models for *Tursiops truncatus* distribution, as run on the full dataset for all years, years 1999-2009, and years 2010-2019. First three rows display results utilizing Negative Binomial distribution, while following three rows utilize Tweedie distribution. Variables that are statistically significant have names marked in blue. All vertical axes indicate the probability of dolphin occurrence on a scale of 0 to 1 (except instances where scale is minimized for clarity), while horizontal axes are presented in the units of the relevant explanatory variable, and above the horizontal axis are markings, indicating number of non-zero observations at that value of explanatory variable. Gray shaded areas represent 95% Confidence Interval.

Full Dataset - Negative Binomial								
All Years Depth	All Years Shore	All Years Slope	All Years SST	All Years Desal	All Years Nutrients	**All Years Searching**	All Years Bottom	All Years Structure
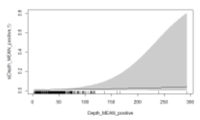	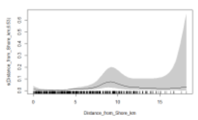	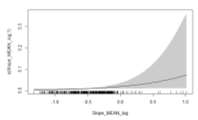	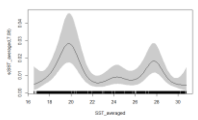	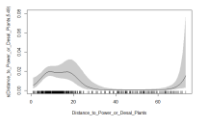		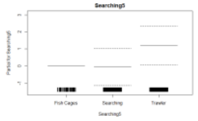	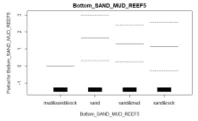	
1999-2009 Depth	1999-2009 Shore	1999-2009 Slope	1999-2009 SST	1999-2009 Desal	1999-2009 Nutrients	**1999-2009 Searching**	1999-2009 Bottom	1999-2009 Structure
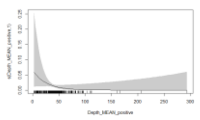	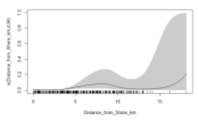	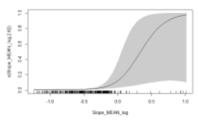	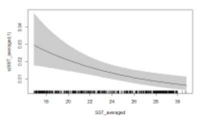	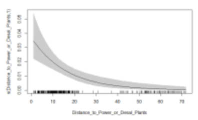		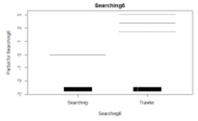	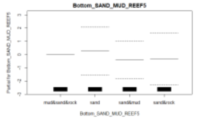	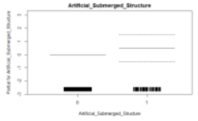
2010-2019 Depth	2010-2019 Shore	2010-2019 Slope	2010-2019 SST	2010-2019 Desal	2010-2019 Nutrients	**2010-2019 Searching**	2010-2019 Bottom	2010-2019 Structure
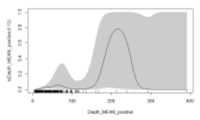	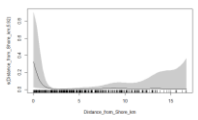		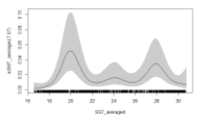	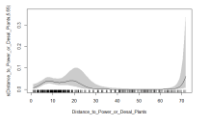	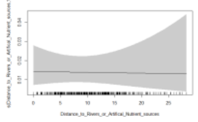	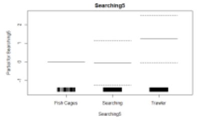		
Full Dataset - Tweedie								
All Years Depth	All Years Shore	All Years Slope	All Years SST	All Years Desal	All Years Nutrients	**All Years Searching**	All Years Bottom	All Years Structure
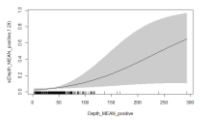	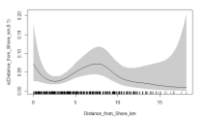		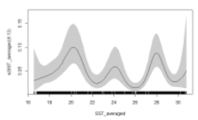	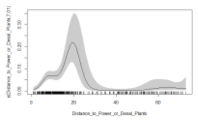	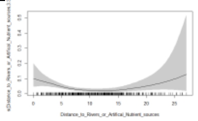	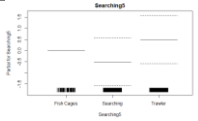		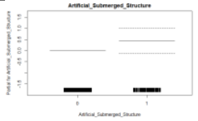
1999-2009 Depth	1999-2009 Shore	1999-2009 Slope	1999-2009 SST	1999-2009 Desal	1999-2009 Nutrients	**1999-2009 Searching**	1999-2009 Bottom	1999-2009 Structure
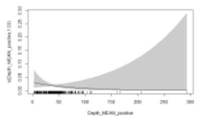	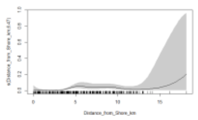	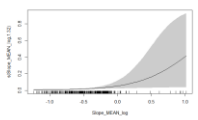	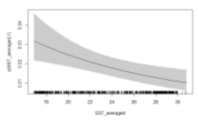	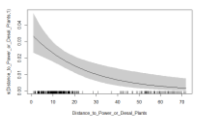		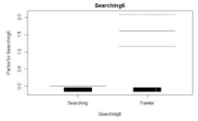		
2010-2019 Depth	2010-2019 Shore	2010-2019 Slope	2010-2019 SST	2010-2019 Desal	2010-2019 Nutrients	**2010-2019 Searching**	2010-2019 Bottom	2010-2019 Structure
	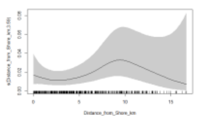		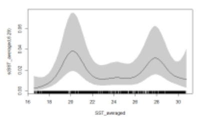	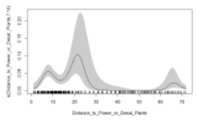		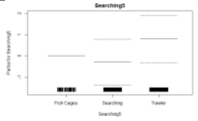		

* Depth = Depth (m), Shore = Distance to Shore (km), Slope = log(Slope), SST = Sea Surface Temperature (°C), Desal = Distance to Desalination or Power Plants (km), Nutrients = Distance to Rivers or Artificial Nutrient Sources (km), Searching = Searching (randomized)/Trawler (in vicinity)/Fish Cages (in vicinity), Bottom = Type of substrate (mud/sand/rock), Structure = Artificial Submerged Structure (present/not present).

Table S7. Final models for *Tursiops truncatus* distribution, as run on the ‘Hot Season’ dataset for all years, years 1999-2009, and years 2010-2019. First three rows display results utilizing Negative Binomial distribution, while following three rows utilize Tweedie distribution. Variables that are statistically significant have names marked in blue. All vertical axes indicate the probability of dolphin occurrence on a scale of 0 to 1 (except instances where scale is minimized for clarity), while horizontal axes are presented in the units of the relevant explanatory variable, and above the horizontal axis are markings, indicating number of non-zero observations at that value of explanatory variable. Gray shaded areas represent 95% Confidence Interval.

Hot Season – Negative Binomial								
All Years Depth	All Years Shore	All Years Slope	All Years SST	All Years Desal	All Years Nutrients	**All Years Searching**	All Years Bottom	All Years Structure
	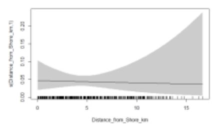		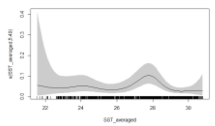	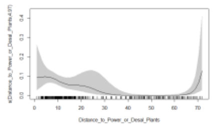		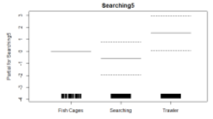	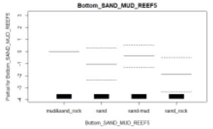	
1999-2009 Depth	1999-2009 Shore	1999-2009 Slope	1999-2009 SST	1999-2009 Desal	1999-2009 Nutrients	**1999-2009 Searching**	1999-2009 Bottom	1999-2009 Structure
	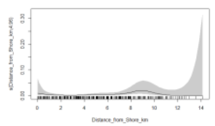		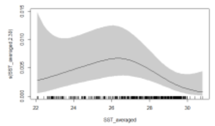		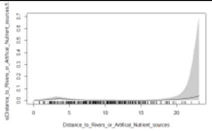	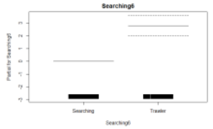		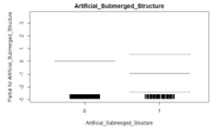
2010-2019 Depth	2010-2019 Shore	2010-2019 Slope	2010-2019 SST	2010-2019 Desal	2010-2019 Nutrients	**2010-2019 Searching**	2010-2019 Bottom	2010-2019 Structure
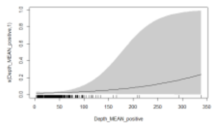	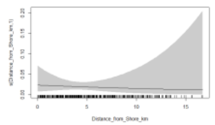		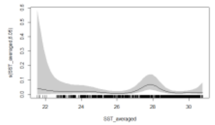	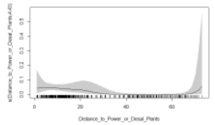		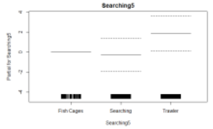	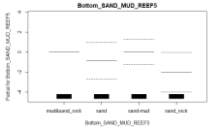	
Hot Season - Tweedie								
All Years Depth	All Years Shore	All Years Slope	All Years SST	All Years Desal	All Years Nutrients	**All Years Searching**	All Years Bottom	All Years Structure
				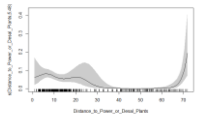		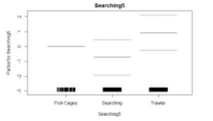	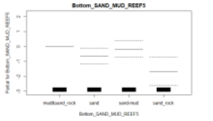	
1999-2009 Depth	1999-2009 Shore	1999-2009 Slope	1999-2009 SST	1999-2009 Desal	1999-2009 Nutrients	**1999-2009 Searching**	1999-2009 Bottom	1999-2009 Structure
						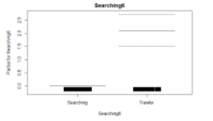		
2010-2019 Depth	2010-2019 Shore	2010-2019 Slope	2010-2019 SST	2010-2019 Desal	2010-2019 Nutrients	**2010-2019 Searching**	2010-2019 Bottom	2010-2019 Structure
				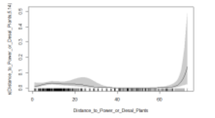		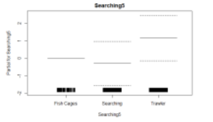		

* Depth = Depth (m), Shore = Distance to Shore (km), Slope = log(Slope), SST = Sea Surface Temperature (°C), Desal = Distance to Desalination or Power Plants (km), Nutrients = Distance to Rivers or Artificial Nutrient Sources (km), Searching = Searching (randomized)/Trawler (in vicinity)/Fish Cages (in vicinity), Bottom = Type of substrate (mud/sand/rock), Structure = Artificial Submerged Structure (present/not present).

Table S8. Final models for *Tursiops truncatus* distribution, as run on the ‘Cold Season’ dataset for all years, years 1999-2009, and years 2010-2019. First three rows display results utilizing Negative Binomial distribution, while following three rows utilize Tweedie distribution. Variables that are statistically significant have names marked in blue. All vertical axes indicate the probability of dolphin occurrence on a scale of 0 to 1 (except instances where scale is minimized for clarity), while horizontal axes are presented in the units of the relevant explanatory variable, and above the horizontal axis are markings, indicating number of non-zero observations at that value of explanatory variable. Gray shaded areas represent 95% Confidence Interval.

Cold Season – Negative Binomial								
All Years Depth	All Years Shore	All Years Slope	All Years SST	All Years Desal	All Years Nutrients	**All Years Searching**	All Years Bottom	All Years Structure
	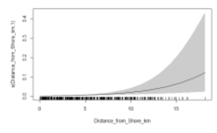	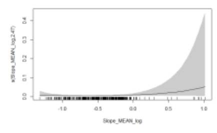	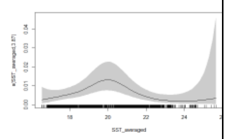	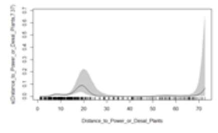	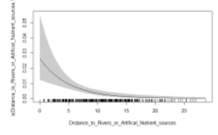	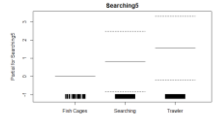		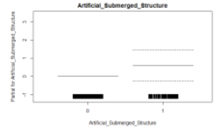
1999-2009 Depth	1999-2009 Shore	1999-2009 Slope	1999-2009 SST	1999-2009 Desal	1999-2009 Nutrients	**1999-2009 Searching**	1999-2009 Bottom	1999-2009 Structure
	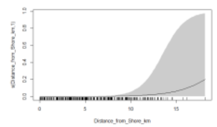	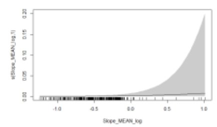		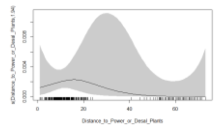	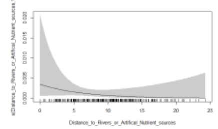	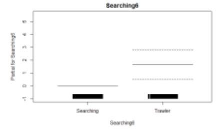	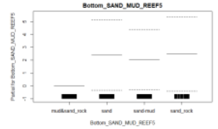	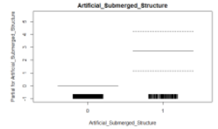
2010-2019 Depth	2010-2019 Shore	2010-2019 Slope	2010-2019 SST	2010-2019 Desal	2010-2019 Nutrients	**2010-2019 Searching**	2010-2019 Bottom	2010-2019 Structure
			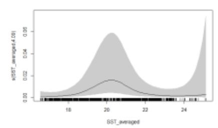	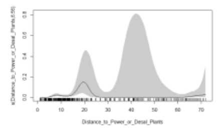	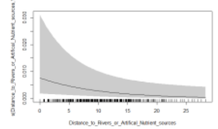	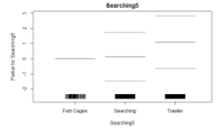		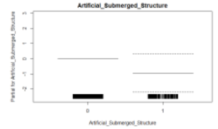
Cold Season - Tweedie								
All Years Depth	All Years Shore	All Years Slope	All Years SST	All Years Desal	All Years Nutrients	**All Years Searching**	All Years Bottom	All Years Structure
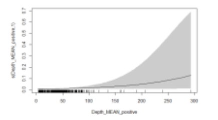			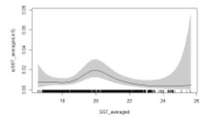	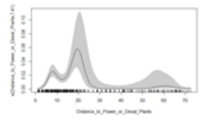		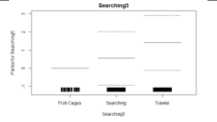		
1999-2009 Depth	1999-2009 Shore	1999-2009 Slope	1999-2009 SST	1999-2009 Desal	1999-2009 Nutrients	**1999-2009 Searching**	1999-2009 Bottom	1999-2009 Structure
						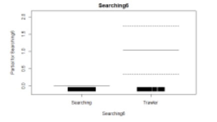		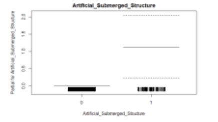
2010-2019 Depth	2010-2019 Shore	2010-2019 Slope	2010-2019 SST	2010-2019 Desal	2010-2019 Nutrients	**2010-2019 Searching**	2010-2019 Bottom	2010-2019 Structure
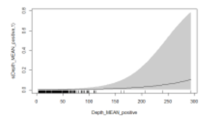	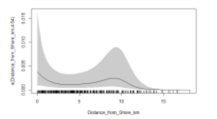			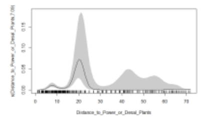		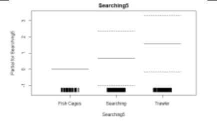		

* Depth = Depth (m), Shore = Distance to Shore (km), Slope = log(Slope), SST = Sea Surface Temperature (°C), Desal = Distance to Desalination or Power Plants (km), Nutrients = Distance to Rivers or Artificial Nutrient Sources (km), Searching = Searching (randomized)/Trawler (in vicinity)/Fish Cages (in vicinity), Bottom = Type of substrate (mud/sand/rock), Structure = Artificial Submerged Structure (present/not present).

Table S9. Final models for *Tursiops truncatus* distribution, as run on the spatial subsets (Areas 1/2/3/4/5) for all years, years 1999-2009, and years 2010-2019. First three rows in each section displays results utilizing Negative Binomial distribution, while following three rows utilize Tweedie distribution. Variables that are statistically significant have names marked in blue. All vertical axes indicate the probability of dolphin occurrence on a scale of 0 to 1 (except instances where scale is minimized for clarity), while horizontal axes are presented in the units of the relevant explanatory variable, and above the horizontal axis are markings, indicating number of non-zero observations at that value of explanatory variable. Gray shaded areas represent 95% Confidence Interval.

Area 1 – Negative Binomial								
All Years Depth	All Years Shore	All Years Slope	All Years SST	All Years Desal	All Years Nutrients	**All Years Searching**	All Years Bottom	All Years Structure
				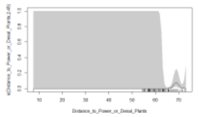				
1999-2009 Depth	1999-2009 Shore	1999-2009 Slope	1999-2009 SST	1999-2009 Desal	1999-2009 Nutrients	**1999-2009 Searching**	1999-2009 Bottom	1999-2009 Structure
			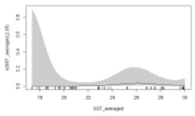					
2010-2019 Depth	2010-2019 Shore	2010-2019 Slope	2010-2019 SST	2010-2019 Desal	2010-2019 Nutrients	**2010-2019 Searching**	2010-2019 Bottom	2010-2019 Structure
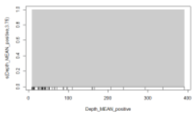				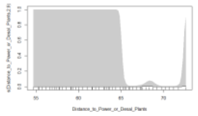		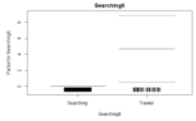		
Area 1 - Tweedie								
All Years Depth	All Years Shore	All Years Slope	All Years SST	All Years Desal	All Years Nutrients	**All Years Searching**	All Years Bottom	All Years Structure
				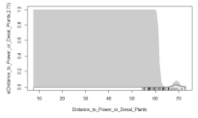		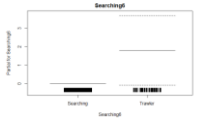		
1999-2009 Depth	1999-2009 Shore	1999-2009 Slope	1999-2009 SST	1999-2009 Desal	1999-2009 Nutrients	**1999-2009 Searching**	1999-2009 Bottom	1999-2009 Structure
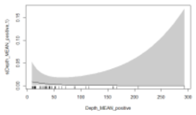		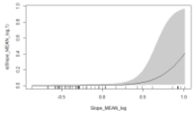						
2010-2019 Depth	2010-2019 Shore	2010-2019 Slope	2010-2019 SST	2010-2019 Desal	2010-2019 Nutrients	**2010-2019 Searching**	2010-2019 Bottom	2010-2019 Structure
				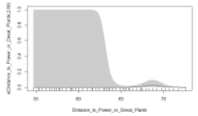		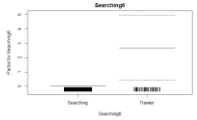		

Area 3 – Negative Binomial								
All Years Depth	All Years Shore	All Years Slope	All Years SST	All Years Desal	All Years Nutrients	**All Years Searching**	All Years Bottom	All Years Structure
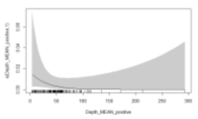				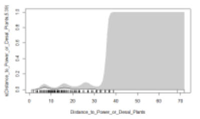		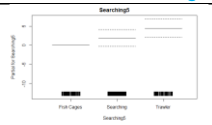	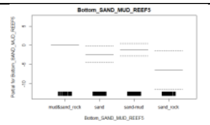	
1999-2009 Depth	1999-2009 Shore	1999-2009 Slope	1999-2009 SST	1999-2009 Desal	1999-2009 Nutrients	**1999-2009 Searching**	1999-2009 Bottom	1999-2009 Structure
						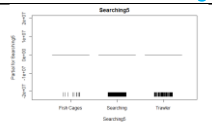	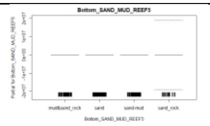	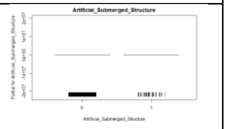
2010-2019 Depth	2010-2019 Shore	2010-2019 Slope	2010-2019 SST	2010-2019 Desal	2010-2019 Nutrients	**2010-2019 Searching**	2010-2019 Bottom	2010-2019 Structure
			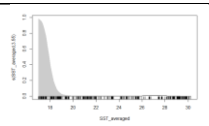				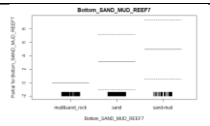	

Area 3 - Tweedie								
All Years Depth	All Years Shore	All Years Slope	All Years SST	All Years Desal	All Years Nutrients	**All Years Searching**	All Years Bottom	All Years Structure
				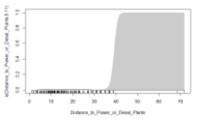		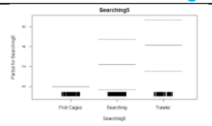		
1999-2009 Depth	1999-2009 Shore	1999-2009 Slope	1999-2009 SST	1999-2009 Desal	1999-2009 Nutrients	**1999-2009 Searching**	1999-2009 Bottom	1999-2009 Structure
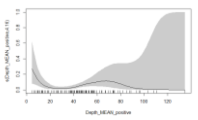						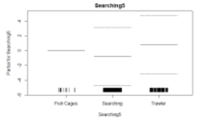		
2010-2019 Depth	2010-2019 Shore	2010-2019 Slope	2010-2019 SST	2010-2019 Desal	2010-2019 Nutrients	**2010-2019 Searching**	2010-2019 Bottom	2010-2019 Structure
		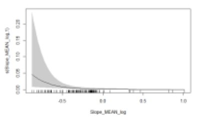						

Area 4 – Negative Binomial								
All Years Depth	All Years Shore	All Years Slope	All Years SST	All Years Desal	All Years Nutrients	**All Years Searching**	All Years Bottom	All Years Structure
	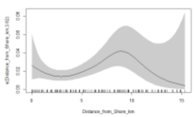		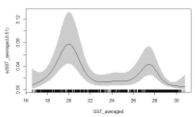			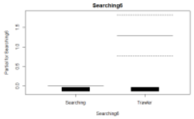		
1999-2009 Depth	1999-2009 Shore	1999-2009 Slope	1999-2009 SST	1999-2009 Desal	1999-2009 Nutrients	**1999-2009 Searching**	1999-2009 Bottom	1999-2009 Structure
			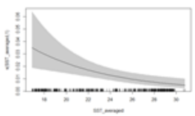			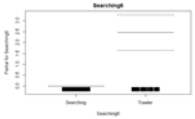		
2010-2019 Depth	2010-2019 Shore	2010-2019 Slope	2010-2019 SST	2010-2019 Desal	2010-2019 Nutrients	**2010-2019 Searching**	2010-2019 Bottom	2010-2019 Structure
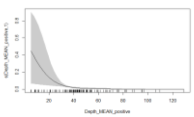	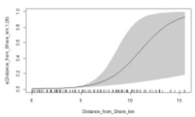		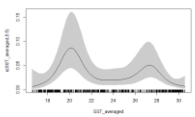			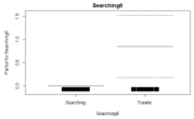		
Area 4 - Tweedie								
All Years Depth	All Years Shore	All Years Slope	All Years SST	All Years Desal	All Years Nutrients	**All Years Searching**	All Years Bottom	All Years Structure
			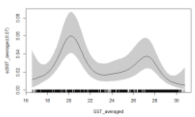			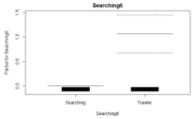		
1999-2009 Depth	1999-2009 Shore	1999-2009 Slope	1999-2009 SST	1999-2009 Desal	1999-2009 Nutrients	**1999-2009 Searching**	1999-2009 Bottom	1999-2009 Structure
						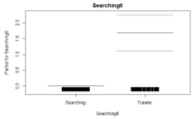		
2010-2019 Depth	2010-2019 Shore	2010-2019 Slope	2010-2019 SST	2010-2019 Desal	2010-2019 Nutrients	**2010-2019 Searching**	2010-2019 Bottom	2010-2019 Structure
				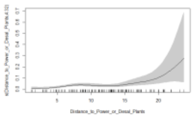		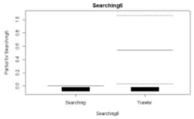		

Area 5 - Negative Binomial								
All Years Depth	All Years Shore	All Years Slope	All Years SST	All Years Desal	All Years Nutrients	**All Years Searching**	All Years Bottom	All Years Structure
						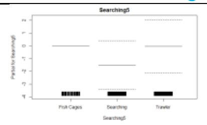		
1999-2009 Depth	1999-2009 Shore	1999-2009 Slope	1999-2009 SST	1999-2009 Desal	1999-2009 Nutrients	**1999-2009 Searching**	1999-2009 Bottom	1999-2009 Structure
			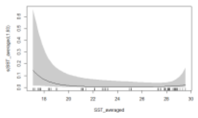					
2010-2019 Depth	2010-2019 Shore	2010-2019 Slope	2010-2019 SST	2010-2019 Desal	2010-2019 Nutrients	**2010-2019 Searching**	2010-2019 Bottom	2010-2019 Structure
						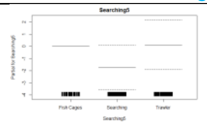		
Area 5 - Tweedie								
All Years Depth	All Years Shore	All Years Slope	All Years SST	All Years Desal	All Years Nutrients	**All Years Searching**	All Years Bottom	All Years Structure
						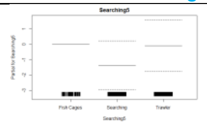		
1999-2009 Depth	1999-2009 Shore	1999-2009 Slope	1999-2009 SST	1999-2009 Desal	1999-2009 Nutrients	**1999-2009 Searching**	1999-2009 Bottom	1999-2009 Structure
			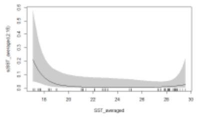					
2010-2019 Depth	2010-2019 Shore	2010-2019 Slope	2010-2019 SST	2010-2019 Desal	2010-2019 Nutrients	**2010-2019 Searching**	2010-2019 Bottom	2010-2019 Structure
			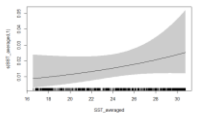					

* Depth = Depth (m), Shore = Distance to Shore (km), Slope = log(Slope), SST = Sea Surface Temperature (°C), Desal = Distance to Desalination or Power Plants (km), Nutrients = Distance to Rivers or Artificial Nutrient Sources (km), Searching = Searching (randomized)/Trawler (in vicinity)/Fish Cages (in vicinity), Bottom = Type of substrate (mud/sand/rock), Structure = Artificial Submerged Structure (present/not present).

Table S10. Summary of 'trawler-excluded' models for each spatial section. Information includes number of sightings within the subset, number of zero values, deviance explained for both Negative Binomial and Tweedie models, and names of explanatory variables for both Negative Binomial and Tweedie models.

Model name	Searching Dolphin Observations	Searching Zero	Trawler Dolphin Observations	Trawler Zero	Fish Cages Dolphin Observations	Fish Cages Zero	% Deviance Explained (NB)	Variable names (NB)	% Deviance Explained (TW)	Variable names (TW)
Sec 3 & 4 & 5, All Years	125	15715	139	3494	6	786	13	DEPTH + DESAL + SST + WRECK	19	DEPTH + SHORE + DESAL + SST
Sec 3, All Years	25	3491	17	366	1	489	33	DESAL + BOTTOM + DEPTH	9	SHORE
Sec 4, All Years	76	7502	96	2119	0	0	21	SST + DEPTH + BOTTOM	2	DESAL
Sec 5, All Years	24	4722	26	1009	5	297	14	NUTRIENTS + DEPTH + DESAL + SST	9	NUTRIENTS + SST

Table S11. Final models for *Tursiops truncatus* distribution, as run on the 'trawler-excluded' dataset (Areas 3&4&5/3/4/5) for all years, years 1999-2009, and years 2010-2019. Results utilizing Negative Binomial distribution are displayed, followed by results utilizing Tweedie distribution. Variables that are statistically significant have names marked in blue. All vertical axes indicate the probability of dolphin occurrence on a scale of 0 to 1 (except instances where scale is minimized for clarity), while horizontal axes are presented in the units of the relevant explanatory variable, and above the horizontal axis are markings, indicating number of non-zero observations at that value of explanatory variable. Gray shaded areas represent 95% Confidence Interval.

Trawler Excluded – Negative Binomial							
Sec 3,4,5 Depth	Sec 3,4,5 Shore	Sec 3,4,5 Slope	Sec 3,4,5 SST	Sec 3,4,5 Desal	Sec 3,4,5 Nutrients	Sec 3,4,5 Bottom	Sec 3,4,5 Structure
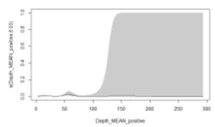			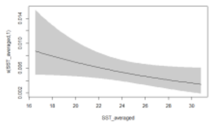	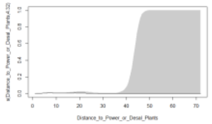			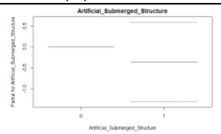
Sec 3 Depth	Sec 3 Shore	Sec 3 Slope	Sec 3 SST	Sec 3 Desal	Sec 3 Nutrients	Sec 3 Bottom	Sec 3 Structure
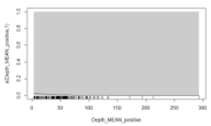				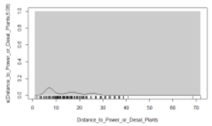		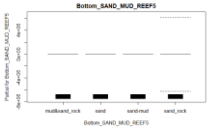	
Sec 4 Depth	Sec 4 Shore	Sec 4 Slope	Sec 4 SST	Sec 4 Desal	Sec 4 Nutrients	Sec 4 Bottom	Sec 4 Structure
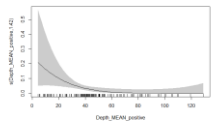			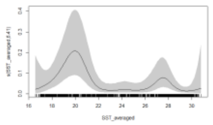			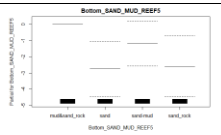	
Sec 5 Depth	Sec 5 Shore	Sec 5 Slope	Sec 5 SST	Sec 5 Desal	Sec 5 Nutrients	Sec 5 Bottom	Sec 5 Structure
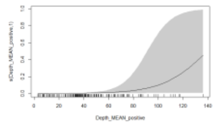			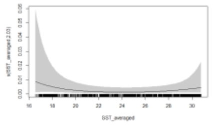	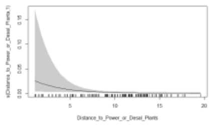	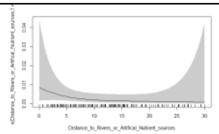		
Trawler Excluded - Tweedie							
Sec 3,4,5 Depth	Sec 3,4,5 Shore	Sec 3,4,5 Slope	Sec 3,4,5 SST	Sec 3,4,5 Desal	Sec 3,4,5 Nutrients	Sec 3,4,5 Bottom	Sec 3,4,5 Structure

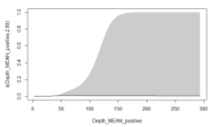	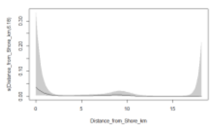		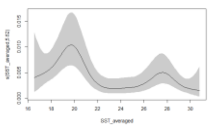	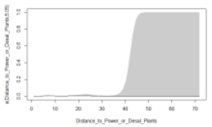			
Sec 3 Depth	Sec 3 Shore	Sec 3 Slope	Sec 3 SST	Sec 3 Desal	Sec 3 Nutrients	Sec 3 Bottom	Sec 3 Structure
	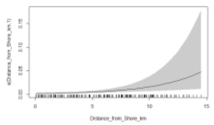						
Sec 4 Depth	Sec 4 Shore	Sec 4 Slope	Sec 4 SST	Sec 4 Desal	Sec 4 Nutrients	Sec 4 Bottom	Sec 4 Structure
				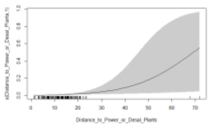			
Sec 5 Depth	Sec 5 Shore	Sec 5 Slope	Sec 5 SST	Sec 5 Desal	Sec 5 Nutrients	Sec 5 Bottom	Sec 5 Structure
			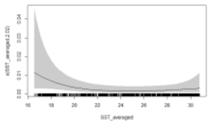		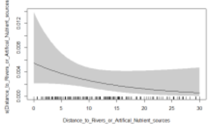		

* Depth = Depth (m), Shore = Distance to Shore (km), Slope = log(Slope), SST = Sea Surface Temperature (°C), Desal = Distance to Desalination or Power Plants (km), Nutrients = Distance to Rivers or Artificial Nutrient Sources (km), Searching = Searching (randomized)/Trawler (in vicinity)/Fish Cages (in vicinity), Bottom = Type of substrate (mud/sand/rock), Structure = Artificial Submerged Structure (present/not present).

Table S12. Summary of models from the three *Delphinus delphis* subsets. Information includes number of sightings within the subset, number of zero values, deviance explained for both Negative Binomial and Tweedie models, and names of explanatory variables for both Negative Binomial and Tweedie models.

Model name	N dolphin Observations	N Zeros	% Non-zero	% Deviance Explained (Negative Binomial)	Variable names (Negative Binomial)	% Deviance Explained (Tweedie)	Variable names (Tweedie)
Subset 2009<br>Full Dataset	40	1012	3.95	38	BOTTOM + SHORE + DEPTH + WRECK + SEARCHING	16	DEPTH
Subset 2016<br>Full Dataset	36	1012	3.56	41	DEPTH + Lat, Lon + SHORE + DESAL	19	DEPTH
Subset 2016, Area 5<br>Full Dataset	35	1032	3.39	29	SHORE + SEARCHING	19	DEPTH

Table S13. Final models for *Delphinus delphis* distribution, as run on three subsets. Results utilizing Negative Binomial distribution are displayed, followed by results utilizing Tweedie distribution. Variables that are statistically significant have names marked in blue. All vertical axes indicate the probability of dolphin occurrence on a scale of 0 to 1 (except instances where scale is minimized for clarity), while horizontal axes are presented in the units of the relevant explanatory variable, and above the horizontal axis are markings, indicating number of non-zero observations at that value of explanatory variable. Gray shaded areas represent 95% Confidence Interval.

Temporal Subsets - Negative Binomial								
Subset 2009 - Depth	Subset 2009 - Shore	Subset 2009 - Lat, Lon	Subset 2009 - SST	Subset 2009 - Desal	Subset 2009 - Nutrients	Subset 2009 - Searching	Subset 2009 - Bottom	Subset 2009 - Structure
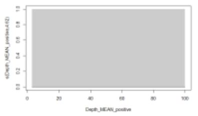	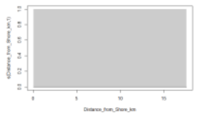					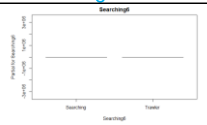	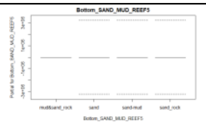	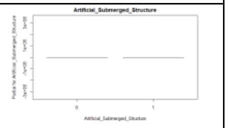
Subset 2016 - Depth	Subset 2016 - Shore	Subset 2016 - Lat, Lon	Subset 2016 - SST	Subset 2016 - Desal	Subset 2016 - Nutrients	Subset 2016 - Searching	Subset 2016 - Bottom	Subset 2016 - Structure
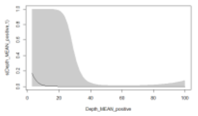	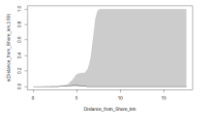	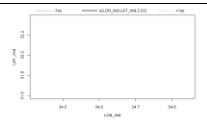		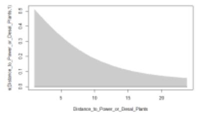				
Subset 2016, Area 5 - Depth	Subset 2016, Area 5 - Shore	Subset 2016, Area 5 - Lat, Lon	Subset 2016, Area 5 - SST	Subset 2016, Area 5 - Desal	Subset 2016, Area 5 - Nutrients	Subset 2016, Area 5 - Searching	Subset 2016, Area 5 - Bottom	Subset 2016, Area 5 - Structure
	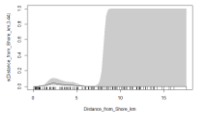					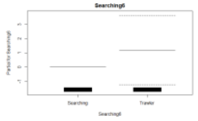		
Temporal Subsets - Tweedie								
Subset 2009 - Depth	Subset 2009 - Shore	Subset 2009 - Lat, Lon	Subset 2009 - SST	Subset 2009 - Desal	Subset 2009 - Nutrients	Subset 2009 - Searching	Subset 2009 - Bottom	Subset 2009 - Structure
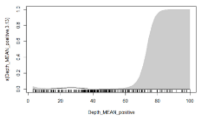								
Subset 2016 - Depth	Subset 2016 - Shore	Subset 2016 - Lat, Lon	Subset 2016 - SST	Subset 2016 - Desal	Subset 2016 - Nutrients	Subset 2016 - Searching	Subset 2016 - Bottom	Subset 2016 - Structure
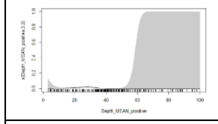								
Subset 2016, Area 5 - Depth	Subset 2016, Area 5 - Shore	Subset 2016, Area 5 - Lat, Lon	Subset 2016, Area 5 - SST	Subset 2016, Area 5 - Desal	Subset 2016, Area 5 - Nutrients	Subset 2016, Area 5 - Searching	Subset 2016, Area 5 - Bottom	Subset 2016, Area 5 - Structure
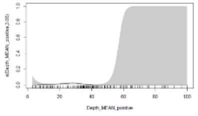								

* Depth = Depth (m), Shore = Distance to Shore (km), Slope = log(Slope), SST = Sea Surface Temperature (°C), Desal = Distance to Desalination or Power Plants (km), Nutrients = Distance to Rivers or Artificial Nutrient Sources (km), Searching = Searching (randomized)/Trawler (in vicinity)/Fish Cages (in vicinity), Bottom = Type of substrate (mud/sand/rock), Structure = Artificial Submerged Structure (present/not present).
